# A Pig Model of Ischemic Mitral Regurgitation Induced by Mitral Chordae Tendinae Rupture and Implantation of an Ameroid Constrictor

**DOI:** 10.1371/journal.pone.0111689

**Published:** 2014-12-05

**Authors:** Yong-Chun Cui, Kai Li, Yi Tian, Wei-Min Yuan, Peng Peng, Jian-Zhong Yang, Bao-Jie Zhang, Hui-Dong Zhang, Ai-Li Wu, Yue Tang

**Affiliations:** State Key Laboratory of Cardiovascular Disease, Fuwai Hospital, National Center for Cardiovascular Diseases, Beijing Municipal Key Laboratory for Pre-clinical Evaluation of Cardiovascular Implantation Devices, Chinese Academy of Medical Sciences and Peking Union Medical College, Beijing, 100037, People's Republic of China; Medical University of Graz, Austria

## Abstract

A miniature pig model of ischemic mitral regurgitation (IMR) was developed by posterior mitral chordae tendinae rupture and implantation of an ameroid constrictor. A 2.5-mm ameroid constrictor was placed around the left circumflex coronary artery (LCX) of male Tibetan miniature pigs to induce ischemia, while the posterior mitral chordae tendinae was also ruptured. X-ray coronary angiography, ECG analysis, echocardiography, and magnetic resonance imaging (MRI) were used to evaluate heart structure and function in pigs at baseline and one, two, four and eight weeks after the operation. Blood velocity of the mitral regurgitation was found to be between medium and high levels. Angiographic analyses revealed that the LCX closure was 10–20% at one week, 30–40% at two weeks and 90–100% at four weeks subsequent ameroid constrictor implantation. ECG analysis highlighted an increase in the diameter of the left atria (LA) at two weeks post-operation as well as ischemic changes in the left ventricle (LV) and LA wall at four weeks post-operation. Echocardiography and MRI further detected a gradual increase in LA and LV volumes from two weeks post-operation. LV end diastolic and systolic volumes as well as LA end diastolic and systolic volume were also significantly higher in pig hearts post-operation when compared to baseline. Pathological changes were observed in the heart, which included scar tissue in the ischemic central area of the LV. Transmission electron microscopy highlighted the presence of contraction bands and edema surrounding the ischemia area, including inflammatory cell infiltration within the ischemic area. We have developed a pig model of IMR using the posterior mitral chordae tendineae rupture technique and implantation of an ameroid constrictor. The pathological features of this pig IMR model were found to mimic the natural history and progression of IMR in patients.

## Introduction

Ischemic mitral regurgitation (IMR) is characterized by the backflow of blood from the left ventricle into the left atrium of the heart [1]. IMR is an important clinical cardiovascular problem as it is one of the most common complications associated with myocardial infarction resulting from changes in left ventricular structure and function. According to previous reports, the incidence of IMR can increase to (i) 40% after myocardial infarction, (ii) 20% during coronary artery disease and (iii) 50% during congestive heart failure [2].

The dominate theories for mechanisms underlying IMR include: (i) imbalance of leaflet tethering and closing force, (ii) left ventricular remodeling, (iii) left ventricular dysfunction, (iv) dyssynchrony of the left ventricular electromechanical activity and (v) changes in spatial structure of the annulus [3], [4]. It is still unclear what initiates the degenerative cellular changes in the mitral valve and myocardium that lead to disease. Thus, further studies elucidating the mechanisms involved in the progression of IMR are needed to improve diagnostics and therapies. Furthermore, the lack of a reliable mammalian model to study the underlying mechanisms for IMR progression remains a critical issue in the IMR research field.

IMR is associated with a worse prognosis after myocardial infarction and subsequent revascularization [5], [6]. At present, medical therapies are not effective for IMR. A combination of angiotensin-converting enzyme inhibitors and beta-blockade can indirectly prevent IMR by inhibiting left ventricular remodeling. However, the incidence and severity of IMR cannot be circumvented through this approach [7]. Surgical treatment strategies for IMR also remain limited and ineffective. Mitral valve repair or replacement, restrictive annuloplasty and coronary artery bypass grafting (CABG) have been widely used as surgical methods for IMR treatment for many years [8], [9], but the persistence and recurrence rates of mitral regurgitation remain high in these patients [10].

Since treatments for IMR remain controversial, the field has focused on developing animal models (including chordae tendineae rupture, rapid pacing and ischemia) to study IMR pathophysiology and test therapeutic approaches for IMR [11]. The reported mortality and complication rate within these models remains high and does not represent the natural history of IMR progression in patients [11]. Our study aimed to develop a pig model of IMR using a posterior mitral chordae tendinae rupture technique and implantation of an ameroid constrictor. We show that this model clinically mimics IMR disease features found in patients, while avoiding the lengthy time required to detect disease pathogenesis in patients naturally suffering from coronary heart disease induced by mitral regurgitation. We provide an in-depth characterization of the pathogenesis of IMR within this pig model, which includes the impact on blood flow, heart function and anatomical location of the mitral lesion at different time points post-operation. We provide evidence for a novel pig model of IMR that recapitulates the natural history of IMR similar to patients. We further provide a stable, feasible and reproducible technique to induce IMR with a high success rate. This model can be exploited to test new therapies and explore the pathological mechanisms underlying IMR as well as determine the influence of etiologies found secondary to IMR, such as atrial fibrosis, atrial fibrillation, structural remodeling of myocardium and heart failure.

## Materials and Methods

### Animals

All animal procedures were in full compliance with the guidelines approved by the Animal Welfare and Ethics Committee in Fuwai Hospital, Chinese Academy of Medical Sciences, Beijing, China (permission number of 2012-1-40-BKJ).

Male Tibetan miniature pigs that were 6 months of age (n = 13) and weighed 22–28 kg were obtained from the Beijing Paike biotechnology company. Pigs had free access to autoclaved water and were fed twice a day.

General anesthesia was intramuscularly administered in pigs with ketamine (35 mg/kg) and diazepam (1.5 mg/kg). A 14-French cuffed endotracheal tube was used for intubation in pigs and ventilation was administered at 15 ml/kg and FiO_2_ at 100%. During this time, pigs were maintained with half doses of ketamine (15 mg/kg, i.v. per 1–2 h) and diazepam (0.75 mg/kg, i.v. per 1–2 h). Pigs were placed on their right lateral sides, using sterile techniques. Subsequently, the pericardium was opened at intercostal segments 2 and 3 by performing a small thoracotomy. A pericardial incision was performed and a 2.5-mm ameroid constrictor was placed around the left circumflex coronary artery (LCX), which resulted in myocardial ischemia in a region supplying blood to the LCX (including papillary muscle ischemia). At the same time, a moderate to severe mitral regurgitation was achieved by pulling off the mitral valve chordae tendinae through a mitral strain device. A pocket was sewn on the left atrial appendage and then the mitral strain device was put through a small incision on the left atrial appendage, which was deep within the left ventricle. Guided by epicardial ultrasound and using cutting barbs on the cutting rod ends, we selectively cut the mitral valve chordae behind P2 and P3 of mitral chordae. As a result, moderate to severe mitral regurgitation was induced in the pigs. In the sham group, pigs were treated the same as in the IMR group but did not undergo ameroid ring implantation and injury of the mitral valve. After the surgery, fentanyl (100 ug, IM) was administered as an analgesic. Penicillin (6,400,000 U/day, i.m.), aspirin (100 mg per day for 1 week), hydrochlorothiazide (8 mg per day for 1 week) and potassium citrate (1.46 g twice per day) were also administered to all pigs.

### Conventional X-ray coronary angiography

X-ray angiography was performed on pigs at baseline as well as one, two and four weeks after surgery using standard transfemoral Judkins techniques that were employed after right and left intracoronary administration of 10 ml iopromide (Clarograf; Juste, Madrid, Spain). Cardiac coronary angiograms were obtained by dynamic scanning of the pig heart at a speed of 3 frames per second with an X-ray machine (Philips).

### Electrocardiographic recordings

Electrocardiograms were recorded as previously described [12]–[16]. Three standard bipolar limb lead (I, II, III), electrodes were placed on the cubital and stifle joints of the pig. For chest leads, V1 was located at the right edge of the sternum in the fourth intercostal space; while V2 was located at the left sternal border in the fourth intercostal space. V2 was also located at the midpoint of the connection of V3 and V4. V4 was located at the intersection of the left mid-clavicle line and fifth intercostal space. V5 was found at the left anterior axillary line and at the same level as V4. V6 was located at the left midaxillary line at the same level as V4. The paper speed was 25 mm/sec and sensitivity was 10 mm/mV.

### Echocardiography

Pigs were anesthetized as described above. Echocardiography was performed with a biplane scanner (Philips Sonos 7500) ultrasound system using a 2.5-MHz transducer (Philips Medical Systems, Andover, MA). Ejection fraction (EF), left ventricular end diastolic volume (LVEDV), left ventricular end systolic volume (LVESV) and left ventricular mass were determined using the Cube formula [17], [18]. The regurgitation fraction is the percentage of blood that regurgitates back through the mitral valve into the atrium. It is calculated as the amount of blood regurgitated into the left atrium divided by the stroke volume. Left atrial (LA) volumes were calculated from four chamber views using the modified biplane Simpson's method. For the mitral annulus, a straight line was extrapolated connecting the attachment points of the leaflets to the valve ring. Automatic volumes were calculated using the software package associated with the biplane scanner (Philips Sonos 7500) that we programmed to include the modified Simpson's disc summation method. Maximum LA volume (LA end-diastolic volume [LAEDV]) was measured at ventricular end-systole and the minimum LA volume was measured at atrial end-systole (LA end-systolic volume [LAESV]). The LA appendage and pulmonary veins were excluded from LA volume measurements. Echocardiograms were interpreted by a single cardiologist who was blinded to the animal groups.

### Magnetic resonance imaging of hearts

Magnetic resonance imaging (MRI) was performed in all pigs at baseline as well as one and thirteen weeks post-surgery using a 1.5T whole body imager (Magnetom Sonata scanner, Siemens Medical Solutions, Erlangen, Germany). Specific absorption rates (SAR) were limited to below 2.2 W/kg and the average SAR for the chest was 1.4 W/kg using fast gradient-echo sequences. MRI results were interpreted by a radiologist who was blinded to the animal groups.

### Cardiac troponin I and BNP measurements

Cardiac troponin I (cTnI, 2010-4-HSP, Life Diagnostics, Inc West Chester, PA) and B-natriuretic peptide (BNP, Cat No. PR40079, Bio SWAMP, China) were measured by an enzyme immunoassay that uses pig specific enzyme-linked immunosorbent assay kits. The assay was performed according to the manufacturer's instructions.

### Histology and transmission electron microscopy

Hearts were harvested from pigs and fixed for 24-hours in formalin. Fixed hearts were embedded in paraffin. Serial coronal sections were obtained from the ventricle and evenly distributed into five slides. Cardiac sections were stained with nitro blue tetrazolium (N-BT) for 15 min at room temperature. A HPIAS-1000 medical imaging system (Raise Dragon Science & Technic Co.,Ltd, Beijing, China) was used to measure the infarct area (no NBT staining) and non-infarcted area (NBT staining area). Myocardial infarct sizes and ratios were calculated by dividing myocardial infarct area to total ventricular area. Serial transverse sections were also obtained along the entire length of the embedded hearts. Cardiac sections were stained with hematoxylin and eosin (H&E Sigma, Shanghai, China) and Masson's trichrome stain. The histopathological severity of each heart was determined by scoring the number of inflammatory cells, area of infiltration, presence of interstitial edema, presence of neutrophil infiltration and the size of the necrotic area.

For electron microscopy, hearts were fixed for 1 hour at 4°C in a cacodylate buffer containing 1% osmium tetroxide and subsequently treated with tannic acid as an en bloc stain to enhance the contrast of elastic laminae as described previously [19]. Hearts were subsequently rinsed in water, dehydrated in a graded series of solutions containing ethanol to propylene oxide and subsequently embedded in epoxy resin. Semi-thin sections were obtained and stained with azur II–methylene blue and examined by light microscopy to select for appropriate fields for further analysis. Ultrathin sections were subjected to contrast agents, uranyl acetate and lead citrate and then subsequently examined with a CM Philips transmission electron microscope (KE Electronics, Tofts, United Kingdom).

### Statistical analyses

Data are presented as mean ± standard error of the mean. Statistical analyses were performed using the unpaired student t-test or analysis of variance followed by the Newman-Keuls test. A Chi-Square test was also performed to analyze differences between images. P-values found to be less than 0.05 were considered statistically significant. Statistical analyses were performed with the use of the SPSS 11.0 software (SPSS, Inc, Chicago, IL).

## Results

### Coronary angiography and cardiac electrocardiography in pigs

**Figure 1:**
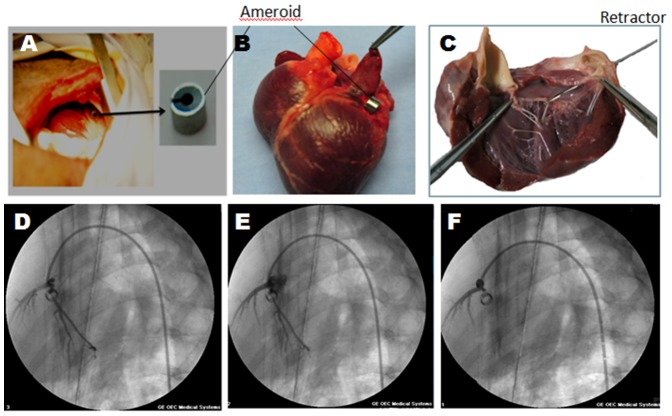
Coronary angiography to detect vessel closure in operated pigs induced by ameroid constrictor. The pericardium was opened and a 2.5-mm ameroid constrictor (A, B) was implanted around the left circumflex coronary artery (C). Representative images show x-ray angiographs from the left coronary system in pig hearts at one (D), two (E), and four (F) weeks, post-surgery. Arrow depicts the ameroid constrictor.

The ameroid constrictor was used to cause vessel closure, which resulted in 10–20% closure at one week, 30–40% closure at two weeks and 90–100% closure at four weeks post-implantation in pigs as well as using the angiographic guide wire as a scale (diameter: 0.21 cm) (Figure 1). ECG parameters were stable in pig hearts for up to four weeks after the surgery. As compared to the baseline, the terminal force of the p wave in lead V1 (PTFv1) was significantly reduced from 0.042±0.031 mm·s to 0.021±0.017 mm·s at four weeks post-surgery in pig hearts. Analysis of leads I, aVL, V5 and V6 displayed signs of ST segment elevation and T-wave inversion. Pathological Q waves were also observed in leads V1 and V2. These leads also revealed ST-segment depression indicative of left ventricular and atrial wall ischemia. The decreased P terminal force v1 (PTFv1) implied increased left atrial diameter and left ventricular diastolic volume load.

### cTnI and BNP level in pigs

**Figure 2:**
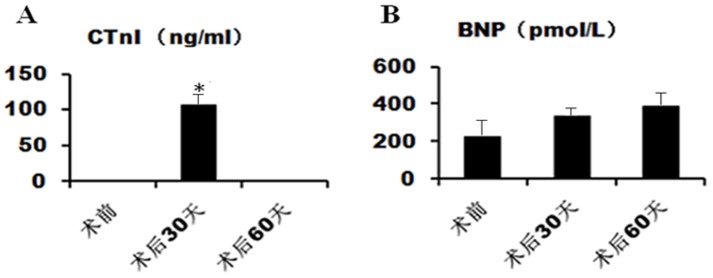
Analysis of cardiac troponin I and B-natriuretic peptide levels in plasma of operated pigs. Analysis of cardiac troponin I (cTnI, A) and B-natriuretic peptide (BNP, B) plasma levels in operated pigs at baseline (a), 30 days (b) and 60 days (c) post-surgery. *P<0.05 versus baseline level.

The cTnI concentration was measured in sera of pigs at baseline as well as 30 and 60 days post-surgery. There was a peak (106.8 ng/ml) in cTnI levels at 30 days after surgery, indicative of the severity of ischemic injury (Figure 2). However, the concentration of BNP was lower than 400 pmol/L at all of the observed time points (Figure 2), suggesting that no decompensation in systolic and diastolic function had occurred.

### Echocardiography in pigs

**Figure 3:**
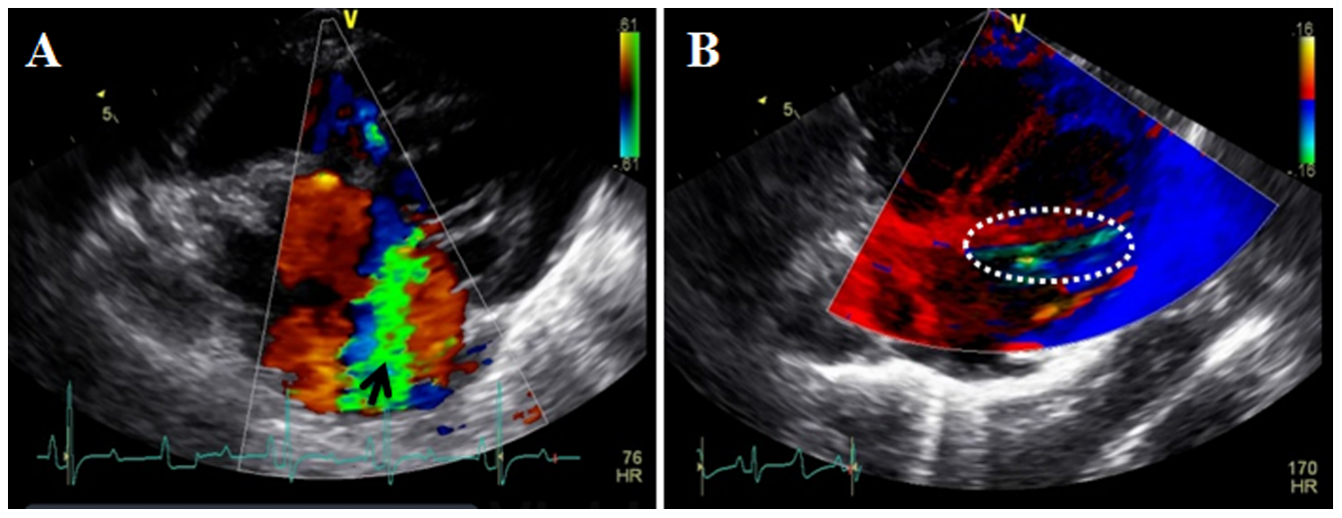
Representative color Doppler views of the pig heart following surgery. A: Representative long-axis view of the heart. The arrow depicts regurgitated blood. B: Motion shifts in the ventricular tissue. Green area depicts the uncoordinated area of myocardial motion.

**Figure 4:**
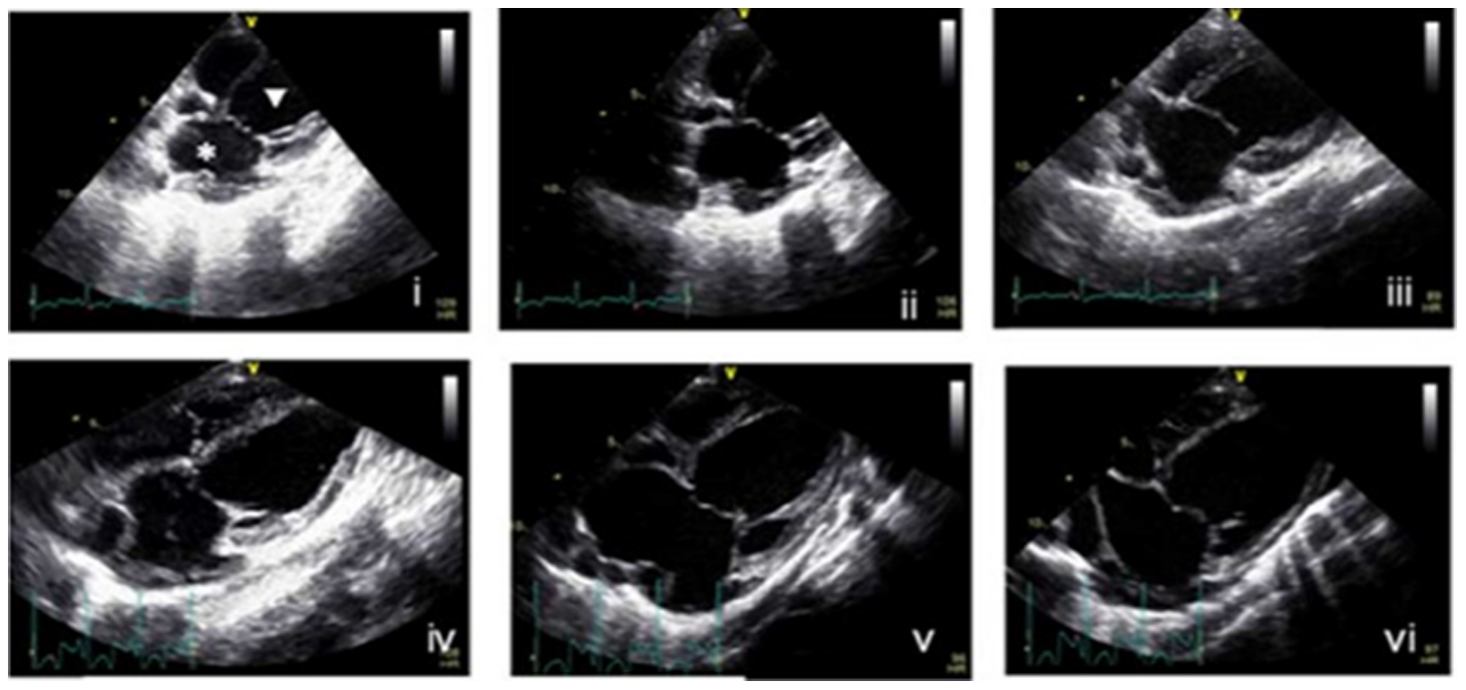
Analysis of left ventricular dimensions in operated pigs by echocardiography. Representative apical long-axis views of the four chambers in the pig heart by two-dimensional echocardiography – (i) before surgery, (ii) immediately after surgery, (iii) one week after surgery, (iv) two weeks after surgery, (v) four weeks after surgery and (vi) eight weeks after surgery. *, left atrial; ▿, left ventricular.

Color Doppler flow imaging was used to detect eccentric “drop-like” jets of mitral valve regurgitation near the left atrial wall in all pigs. The abnormal movement of the mitral muscle could also be detected. We showed that the mitral ring gradually expanded in pig hearts with increasing left ventricular (systolic and diastolic) and atrial volumes (Figure 3A). All of the above changes were indicative of a regurgitation jet that was caused by ischemic mitral regurgitation. Assessment of the regurgitation area (RA), left atrial area (LAA), regurgitation volume (RV) and regurgitation fraction (RF) was obtained using previously established methods [20], [21]. RA and RA/LAA were increased by 30% from baseline, at time points one, two, four, and eight weeks post-surgery (Table 1). The RV was 0.5 ml greater post-surgery, while RF ranged from 30% to 60% and the regurgitation jet velocity was greater than 300 cm/s at all of the observed time points post-surgery. These characteristics were indicative of moderate to severe mitral regurgitation. The RV was also significantly increased at two weeks post-surgery, which related to the gradual closing of the ameroid ring and posterior papillary muscle ischemia. The slight decrease in RA/LAA at two weeks post-surgery could be due to the gradual increase in atrial pressure and area. One week post-surgery, the LVEDV and LVESV were not significantly different from baseline, while the LAESV and LAEDV had significantly increased over baseline values (Table 2). However, four weeks post-surgery, the LVEDV, LVDSV, LAEDV and LAESV were all significantly increased when compared to baseline values, indicating significant left ventricular remodeling. Eight weeks post-surgery, changes to the LAEDV and LAESV were even greater than those of LVEDV and LVESV in pig hearts. In fact, LAEDV and LAESV were found to be three times higher than baseline (Figure 4).

**Table 1:** Regurgitation parameters in operated pig hearts.

	Before surgery	After surgery	One week after surgery	Two weeks after surgery	Four weeks after surgery	Eight weeks after surgery
Regurgitation area (RA cm^2^)	-	1.9±0.6	2.3±0.3	2.6±0.3	2.5±2.2	2.8±0.9
left atrial area (LA A, cm^2^)	4.5±0.3	4.9±0.5	6.3±0.2*	8.3±0.2*	8.6±0.5*	9.2±2.2*
RA/LAA (%)	-	36.5±0.2	37.7±2	31.3±2.2	30.1±3.9	30.4±2.3
Regurgitation volume (RV ml)	-	1.3±0.6	1.9±0.6	5.3±3.6	5.2±4.3	6.0±1.9
Regurgitation fraction (RF %)	-	39.5±0.3	38.7±2	42.5±1.2	46.0±2.6	49.3±3.5
Regurgitation velocity (cm/s)	-	435.7±34.6	331.5±16.1	325.1±28.4	329.8±13.4	414.2±36.8

*P<0.05 vs baseline.

**Table 2:** Cardiac dimensions and function in operated pigs.

	Before surgery	After surgery	One week after surgery	Two weeks after surgery	Four weeks after surgery	Eight weeks after surgery
LVEDV (ml)	29.4±2.2	26.7±3.2	30.8±2.1	37.9±3.4*	44.8±2.8*	48.9±4.5*
LVESV (ml)	8.5±0.7	7.9±0.6	7.7±0.8	9.7±0.5	15.2±1.3*	16.3±1.5*
EF (%)	69.4±2.9	77.8±0.1	73.7±5.9	74.6±2.2	66.0±7.7	65.0±6,4
E/A	1.5±0.8	2.0±0.3	1.8±0.9	1.4±0.4	1.7±0.7	1.6±0.7
LAEDV (ml)	13.4±1.0	22.7±0.6	24.3±2.2*	24.8±1.6*	33.0±2.1*	35.0±3.8*
LAESV (ml)	5.3±0.6	8.1±0.3	8.9±0.2	9.5±0.4	12.0±1.1*	13.1±1.2*

*P<0.05 vs baseline.

LVEDV: Left ventricular end diastolic volume, LVESV: Left ventricular end systolic volume, EF: Ejection fraction, LAEDV: Left atrial end diastolic volume, LAESV: Left atrial end systolic volume.

Although color Doppler analysis indicated structural changes to the heart, both the sV and EF were not significantly different at any of the observed time points (Table 2). The E/A value was also not significantly different between baseline and time points post-surgery. However, the peak speeds of the A and E waves were higher post-surgery than at baseline (P<0.01 and P<0.05, respectively), indicating that the MR might mask diastolic dysfunction in the left ventricle.

### MRI analysis in pigs

**Figure 5:**
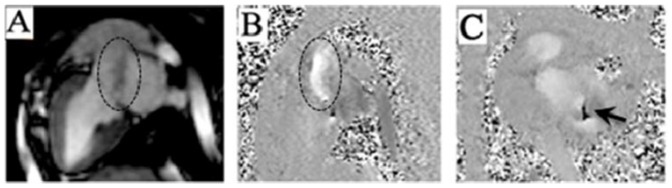
MRI analysis of operated pig hearts. A: Representative long-axis view of the left ventricle; B: Blood flow analysis in the same MRI plane; C: Representative short-axis view of the mitral valve. Arrow depicts regurgitation aperture of the mitral valve. Circle depicts regurgitation of blood.

Cardiac MRI was performed in pig hearts at baseline and eight weeks post-surgery (Figure 5). MRI results indicated a shift in the mitral valve orifice. Systolic regurgitation displayed an eccentric shape while entering the left atrium, which then spread immediately to the left atrium and left atrial posterior wall (Figure 5A and 5B). Regurgitation characteristics observed via MRI were consistent with those observed using color Doppler imaging. The short axis view of the mitral valve indicated that the mitral muscle was dysfunctional (Figure 5C).

### Cardiac pathology in pigs

**Figure 6:**
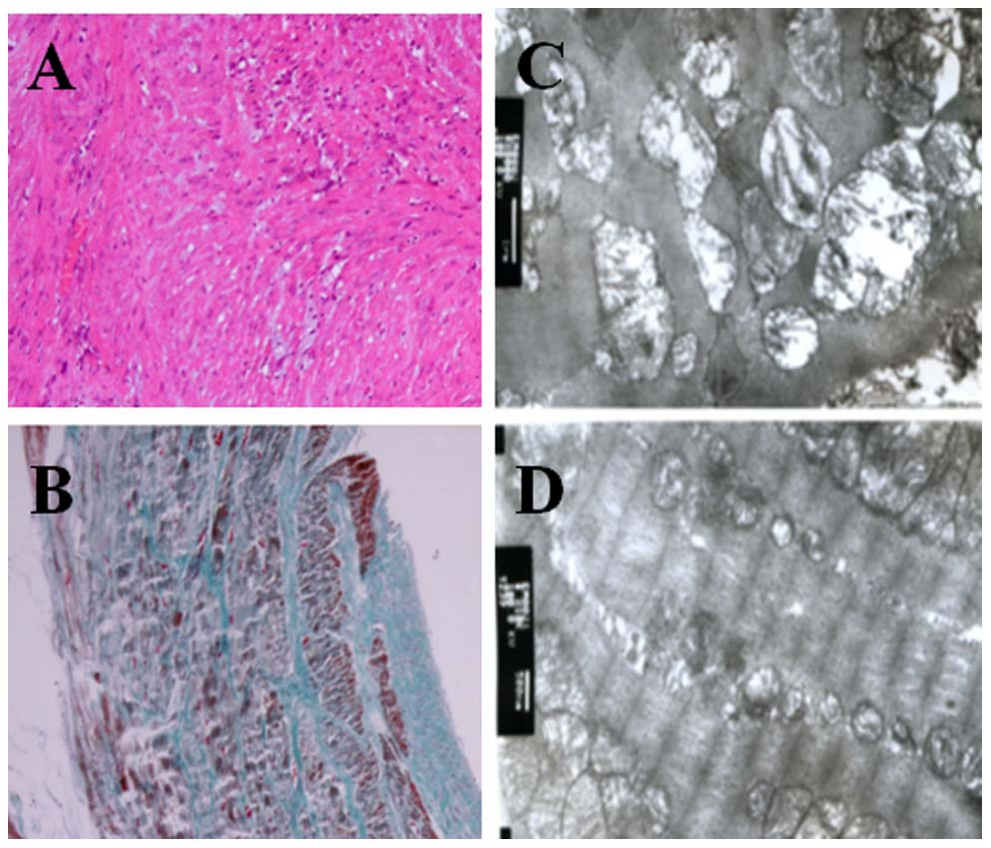
Histopathological analyses of the myocardium in operated pigs. A: H&E analysis of heart sections from operated pigs revealed compensatory thickening of the left ventricle and myocyte disarray. B: Masson trichrome stain of heart sections from operated pigs revealed left atrial fibrosis. Note the significant endocardial fibrosis. C&D: Transmission electron microscopy analysis revealed contraction bands and edema surrounding the ischemic area as well as inflammatory cell infiltration (8000×).

Analyses of pig heart structure post-surgery revealed that the pig hearts displayed chordae tendinae rupture (Grade I or Grade II) of the mitral valve and “jet lesions”, which have been previously described in the left ventricle [22], [23] and could be macroscopically observed in all cases. All of the hearts had undergone significant expansion and increased cardiac sphericity (Figure 6A). H&E and Masson trichrome stain analyses revealed that chronic ischemia and mitral regurgitation caused the cardiac pathological changes, including eccentric ventricular hypertrophy (Figure 6B), fibrosis of the left atrium (Figure 6) and scarring within the ischemic central area of the left ventricle. Transmission electron microscopy further demonstrated the appearance of contraction bands, edema surrounding the ischemic area and inflammatory cell infiltration within the ischemic area. Interestingly, there remained regions of heart muscle that contained an intact nucleus and uniform cytoplasmic staining. Proliferative changes were also retained within the normal (spared) tissue areas (Figure 6C). N-BT staining further revealed that the myocardial infarct area accounted for 7.6±1.8% of the total volume of the ventricle.

## Discussion

IMR is a unique valvular heart disease caused by MR resulting from rupture or extension of the papillary muscle after myocardial infarction. It is different from MR induced from systemic diseases that target the mitral valve such as rheumatic valvular disease and mitral valve prolapse. IMR is a multi-faceted and multi-stage disease that is characterized by local and global left ventricular remodeling [24], [25]. As a result, it is difficult for current models to accurately recapitulate all of the pathological features associated with IMR [26], [27]. Using a posterior mitral chordae tendinae rupture technique and implantation of an ameroid constrictor, we created a miniature pig IMR model. The heart structure and function of the pig model was similar to the natural history of pathogenesis observed in human IMR patients.

Animal models and clinical studies have demonstrated the multifaceted and pathophysiological origins of IMR [28]. The presence of a cardiac-specific troponin in the peripheral blood at levels above normal is a good marker of damage to cardiac muscle cells in cases of myocardial infarction, myocarditis, trauma, cardiac surgery and other cardiac procedures. Troponins start to rise approximately 4–6 h after the onset of acute myocardial infarction and peak at approximately 24 h. They remain elevated for 7–10 days given the long diagnostic window. It is well accepted that troponin levels contribute to the diagnosis and classification of various types of acute coronary syndromes [29]. Troponins can also be sensitively detected by ELISA and can detect low levels of injury in myocardial tissues. In this study, we induced significant ischemia in our pig model of IMR by implanting an ameroid constrictor. ECG analysis indicated an increase in left atrial diameter and ischemic changes in the left ventricle and atrial walls post-surgery. Echocardiography and MRI were able to detect gradual increases in left atrial and ventricular volumes in pigs post-surgery. All of these changes were reminiscent of the disease features associated with IMR in humans. We also show consistently that the level of circulating troponins increased to 106.8 ng/ml at 30 days post-implantation of an ameroid constrictor, implying that the ameroid constrictor had induced the cellular pathological changes. Furthermore, the (i) eccentric “drop-like” jets indicative of mitral regurgitation near the left atrial wall, (ii) abnormal movement of the mitral muscle, and (iii) gradual increase in left ventricular systolic and diastolic volumes as well as left atrial volumes, further indicated that the regurgitation jet was caused by an ischemic mitral regurgitation, a critical feature observed in the natural history of IMR in humans. Moreover, brain natriuretic peptide (BNP), which is a cardiac neurohormone secreted from ventricles in response to ventricular volume expansion and pressure overload, was also assessed in pigs. Blood BNP levels can be used as a biochemical marker for congestive heart failure [30]. We showed that serum BNP levels did not exceed the normal limit until 60 days post-surgery, indicating that no significant changes in left ventricular ejection fraction and cardiac decompensation had occurred. Meanwhile, E/A values as well as sV and EF, were not significantly altered at any of the observed time points in pigs post-surgery. However, the peak speed of the A and E waves were significantly higher than baseline indicating that the MR might have masked diastolic dysfunction in the left ventricle. While our results are encouraging, more work is necessary to improve this miniature pig IMR model. Important limitations of the current study include: (1) the limited number of animals used to confirm the efficiency of our protocol, (2) the necessity of using minimally invasive thoracoscopic surgery to reduce the risk of infection, although there was no infection observed in pigs during our studies, (3) lack of evaluation of hemodynamic parameters (cardiac output, LV and aortic pressures, wedge pressure, etc), and (4) lack of testing the effects of respiratory function on cardiac function parameters. All of these issues are important and future goals of our studies.

## Conclusions

We have developed a novel pig IMR model using a posterior mitral chordae tendinae rupture technique and implantation of an ameroid constrictor. The pathological features of this pig IMR model mimicked the natural evolution of IMR in patients. We present an IMR model that should provide unprecedented opportunities to study pathogenesis and therapeutic interventions for IMR.

## Supporting Information

Table S1 Cardiac troponin I levels in plasma of operated pigs.
(DOC)

Table S2 B-natriuretic peptide levels in plasma of operated pigs.
(DOC)

Table S3 Cardiac dimensions, function and regurgitation parameters in control pig heart. Note: LVEDV: Left ventricular end diastolic volume, LVESV: Left ventricular end systolic volume, EF: Ejection fraction, LAEDV: Left atrial end diastolic volume, LAESV: Left atrial end systolic volume. The same with those in table D-I.
(DOC)

Table S4 ardiac dimensions, function and regurgitation parameters before surgery in operated pig heart.
(DOC)

Table S5 Cardiac dimensions, function and regurgitation parameters immediately after surgery in operated pig heart.
(DOC)

Table S6 Cardiac dimensions, function and regurgitation parameters one week after surgery in operated pig heart.
(DOC)

Table S7 Cardiac dimensions, function and regurgitation parameters two weeks after surgery in operated pig heart.
(DOC)

Table S8 Cardiac dimensions, function and regurgitation parameters four weeks after surgery in operated pig heart.
(DOC)

Table S9 Cardiac dimensions, function and regurgitation parameters eight weeks after surgery in operated pig heart.
(DOC)
