# Supplementary material for: A Pig Model of Ischemic Mitral Regurgitation Induced by Mitral Chordae Tendinae Rupture and Implantation of an Ameroid Constrictor
Source: PLoS One. 2014 Dec 5;9(12):e111689. doi: 10.1371/journal.pone.0111689 (PMC4257529; doi:10.1371/journal.pone.0111689)
Supplement: Table S1 — Cardiac troponin I levels in plasma of operated pigs. (DOC) [file pone.0111689.s001.doc]

**Table S1 Cardiac troponin I levels in plasma of operated pigs (ng/ml)**

|  | pig 1 | pig 2 | pig 3 | pig 4 | pig 5 | pig 6 | pig 7 | pig 8 | pig 9 | pig 10 | pig 11 | pig 12 | pig 13 | mean | SD |
| --- | --- | --- | --- | --- | --- | --- | --- | --- | --- | --- | --- | --- | --- | --- | --- |
| basline | 0.02 | 0.04 | 0.03 | 0.05 | 0.03 | 0.02 | 0.03 | 0.04 | 0.01 | 0.03 | 0.04 | 0.05 | 0.04 | 0.03 | 0.01 |
| 30 d | 102.23 | 125.36 | 78.34 | 120.96 | 98.38 | 115.62 | 122.32 | 115.25 | 96.43 | 85.98 | 89.36 | 99.63 | 103.54 | 104.11 | 14.86 |
| 60 d | 0.05 | 0.04 | 0.08 | 0.02 | 0.03 | 0.02 | 0.05 | 0.06 | 0.07 | 0.04 | 0.03 | 0.06 | 0.08 | 0.05 | 0.02 |
