# Supplementary material for: A Pig Model of Ischemic Mitral Regurgitation Induced by Mitral Chordae Tendinae Rupture and Implantation of an Ameroid Constrictor
Source: PLoS One. 2014 Dec 5;9(12):e111689. doi: 10.1371/journal.pone.0111689 (PMC4257529; doi:10.1371/journal.pone.0111689)
Supplement: Table S2 — B-natriuretic peptide levels in plasma of operated pigs. (DOC) [file pone.0111689.s002.doc]

**Table S2 B-natriuretic peptide levels in plasma of operated pigs (pmol/L)**

|  | pig 1 | pig 2 | pig 3 | pig 4 | pig 5 | pig 6 | pig 7 | pig 8 | pig 9 | pig 10 | pig 11 | pig 12 | pig 13 | mean | SD |
| --- | --- | --- | --- | --- | --- | --- | --- | --- | --- | --- | --- | --- | --- | --- | --- |
| basline | 180.09 | 246.32 | 208.36 | 198.52 | 226.89 | 381.67 | 219.43 | 367.56 | 196.79 | 118.64 | 259.63 | 249.32 | 155.63 | 231.45 | 74.46 |
| 30 d | 325.42 | 400.05 | 286.78 | 375.15 | 296.45 | 315.28 | 298.67 | 387.65 | 250.68 | 261.32 | 397.69 | 390.72 | 287.68 | 328.73 | 54.41 |
| 60 d | 420.36 | 447.65 | 386.52 | 371.12 | 422.32 | 378.46 | 395.15 | 435.31 | 372.34 | 283.25 | 365.15 | 372.38 | 411.76 | 389.37 | 41.81 |
