# Supplementary material for: A Pig Model of Ischemic Mitral Regurgitation Induced by Mitral Chordae Tendinae Rupture and Implantation of an Ameroid Constrictor
Source: PLoS One. 2014 Dec 5;9(12):e111689. doi: 10.1371/journal.pone.0111689 (PMC4257529; doi:10.1371/journal.pone.0111689)
Supplement: Table S3 — Cardiac dimensions, function and regurgitation parameters in control pig heart. Note: LVEDV: Left ventricular end diastolic volume, LVESV: Left ventricular end systolic volume, EF: Ejection fraction, LAEDV: Left atrial end diastolic volume, LAESV: Left atrial end systolic volume. The same with those in table D-I. (DOC) [file pone.0111689.s003.doc]

**Table S3 Cardiac dimensions, function and regurgitation parameters in control pig heart**

Note: LVEDV: Left ventricular end diastolic volume, LVESV: Left ventricular end systolic volume, EF: Ejection fraction, LAEDV: Left atrial end diastolic volume, LAESV: Left atrial end systolic volume. The same with those in table D-I.
