# Supplementary material for: A Pig Model of Ischemic Mitral Regurgitation Induced by Mitral Chordae Tendinae Rupture and Implantation of an Ameroid Constrictor
Source: PLoS One. 2014 Dec 5;9(12):e111689. doi: 10.1371/journal.pone.0111689 (PMC4257529; doi:10.1371/journal.pone.0111689)
Supplement: Table S4 — ardiac dimensions, function and regurgitation parameters before surgery in operated pig heart. (DOC) [file pone.0111689.s004.doc]

**Table S4** Cardiac dimensions, function and regurgitation parameters before surgery in operated pig heart

|  | pig 1 | pig 2 | pig 3 | pig 4 | pig 5 | pig 6 | pig 7 | pig 8 | pig 9 | pig 10 | pig 11 | pig 12 | pig 13 | mean | SD |
| --- | --- | --- | --- | --- | --- | --- | --- | --- | --- | --- | --- | --- | --- | --- | --- |
| Regurgitation area (RA cm^2^) | 0 | 0 | 0 | 0 | 0 | 0 | 0 | 0 | 0 | 0 | 0 | 0 | 0 |  |  |
| left atrial area (LA A , cm^2^) | 4.5 | 4.3 | 4.9 | 4.6 | 4.8 | 4.2 | 3.9 | 4.5 | 4.3 | 4.5 | 3.9 | 5 | 4.4 | 4.4 | 0.3 |
| RA/LAA (%) | 0 | 0 | 0 | 0 | 0 | 0 | 0 | 0 | 0 | 0 | 0 | 0 | 0 |  |  |
| Regurgitation volume (RV ml) | 0 | 0 | 0 | 0 | 0 | 0 | 0 | 0 | 0 | 0 | 0 | 0 | 0 |  |  |
| Regurgitation fraction (RF %) | 0 | 0 | 0 | 0 | 0 | 0 | 0 | 0 | 0 | 0 | 0 | 0 | 0 |  |  |
| Regurgitation velocity (m/s) | 0 | 0 | 0 | 0 | 0 | 0 | 0 | 0 | 0 | 0 | 0 | 0 | 0 |  |  |
| LVEDV (ml) | 24.3 | 32.1 | 29.3 | 31.7 | 30.2 | 27.3 | 29.1 | 30.3 | 29.7 | 29.6 | 27.3 | 32.7 | 29.2 | 29.4 | 2.2 |
| LVESV (ml) | 8.2 | 8.4 | 7.6 | 9.3 | 8.8 | 8.6 | 8.4 | 7.2 | 8.1 | 8.9 | 8.5 | 8.7 | 9.8 | 8.5 | 0.7 |
| EF (%) | 70.0 | 65.0 | 70.0 | 72.0 | 68.0 | 70.0 | 65.0 | 70.0 | 73.0 | 67.0 | 69.0 | 75.0 | 68.0 | 69.4 | 2.9 |
| E/A |  |  |  |  |  |  |  |  |  |  |  |  |  |  |  |
| LAEDV (ml) | 14.4 | 13.3 | 15.3 | 14.7 | 12.7 | 13.4 | 13.3 | 13.0 | 14.2 | 12.5 | 12.0 | 13.1 | 12.7 | 13.4 | 1.0 |
| LAESV (ml) | 4.8 | 4.7 | 5.1 | 5.3 | 6.3 | 4.6 | 4.5 | 5.4 | 6.5 | 5.2 | 4.9 | 5.5 | 6 | 5.3 | 0.6 |
