# Supplementary material for: A Pig Model of Ischemic Mitral Regurgitation Induced by Mitral Chordae Tendinae Rupture and Implantation of an Ameroid Constrictor
Source: PLoS One. 2014 Dec 5;9(12):e111689. doi: 10.1371/journal.pone.0111689 (PMC4257529; doi:10.1371/journal.pone.0111689)
Supplement: Table S5 — Cardiac dimensions, function and regurgitation parameters immediately after surgery in operated pig heart. (DOC) [file pone.0111689.s005.doc]

**Table S5 Cardiac dimensions, function and regurgitation parameters immediately after surgery in operated pig heart**

|  | pig 1 | pig 2 | pig 3 | pig 4 | pig 5 | pig 6 | pig 7 | pig 8 | pig 9 | pig 10 | pig 11 | pig 12 | pig 13 | mean | SD |
| --- | --- | --- | --- | --- | --- | --- | --- | --- | --- | --- | --- | --- | --- | --- | --- |
| Regurgitation area (RA cm^2^) | 2.2 | 1.7 | 2.3 | 2.8 | 1.6 | 1.4 | 1.5 | 3.1 | 1.9 | 1.2 | 1.5 | 2.1 | 1.8 | 1.9 | 0.6 |
| left atrial area (LA A , cm^2^) | 4.9 | 4.4 | 4.9 | 6.2 | 5.5 | 4.8 | 4.6 | 5.2 | 4.9 | 3.9 | 4.9 | 5.1 | 5.0 | 4.9 | 0.5 |
| RA/LAA | 0.4 | 0.4 | 0.5 | 0.5 | 0.3 | 0.3 | 0.3 | 0.6 | 0.4 | 0.3 | 0.3 | 0.4 | 0.4 | 0.4 | 0.1 |
| Regurgitation volume (RV ml) | 1.3 | 1.4 | 1.6 | 2.7 | 1.8 | 0.9 | 1.1 | 1.8 | 0.8 | 1.3 | 0.7 | 1.4 | 0.7 | 1.3 | 0.6 |
| Regurgitation fraction (RF %) | 38.9 | 40.5 | 39.7 | 38.8 | 39.7 | 39.6 | 38.9 | 38.7 | 39.6 | 39.8 | 39.4 | 39.8 | 39.5 | 39.5 | 0.5 |
| Regurgitation velocity (m/s) | 422.0 | 370.0 | 397.0 | 406.0 | 466.0 | 410.0 | 487.0 | 476.0 | 429.0 | 437.0 | 468.0 | 438.0 | 458.6 | 435.7 | 34.6 |
| LVEDV (ml) | 30.8 | 32.6 | 27.1 | 28.4 | 24.8 | 27.3 | 26.9 | 28.5 | 24.6 | 26.6 | 22.6 | 20.5 | 26.2 | 26.7 | 3.2 |
| LVESV (ml) | 8.6 | 7.2 | 7.4 | 8.1 | 9.6 | 8.2 | 7.7 | 8.2 | 7.6 | 7.9 | 7.4 | 7.5 | 7.8 | 7.9 | 0.6 |
| EF (%) | 76.7 | 77.2 | 78.5 | 77.0 | 76.4 | 78.2 | 76.6 | 79.4 | 77.3 | 77.9 | 80.6 | 76.6 | 79.0 | 77.8 | 1.3 |
| E/A | 1.8 | 1.9 | 2.2 | 1.7 | 1.8 | 1.6 | 2.1 | 2.4 | 1.8 | 2.6 | 1.9 | 2.5 | 2.1 | 2.0 | 0.3 |
| LAEDV (ml) | 22.6 | 21.8 | 23.7 | 22.5 | 22.6 | 23.1 | 21.4 | 23.2 | 22.4 | 23.0 | 22.8 | 23.2 | 23.3 | 22.7 | 0.6 |
| LAESV (ml) | 8.6 | 7.9 | 7.9 | 8.2 | 8.6 | 7.9 | 8.3 | 8.0 | 8.2 | 8.4 | 7.5 | 7.9 | 8.3 | 8.1 | 0.3 |
