# Supplementary material for: A Pig Model of Ischemic Mitral Regurgitation Induced by Mitral Chordae Tendinae Rupture and Implantation of an Ameroid Constrictor
Source: PLoS One. 2014 Dec 5;9(12):e111689. doi: 10.1371/journal.pone.0111689 (PMC4257529; doi:10.1371/journal.pone.0111689)
Supplement: Table S6 — Cardiac dimensions, function and regurgitation parameters one week after surgery in operated pig heart. (DOC) [file pone.0111689.s006.doc]

**Table S6 Cardiac dimensions, function and regurgitation parameters one week after surgery in operated pig heart**

|  | | | | | | | | | | | | | | | |
| --- | --- | --- | --- | --- | --- | --- | --- | --- | --- | --- | --- | --- | --- | --- | --- |
|  | pig 1 | pig 2 | pig 3 | pig 4 | pig 5 | pig 6 | pig 7 | pig 8 | pig 9 | pig 10 | pig 11 | pig 12 | pig 13 | mean | SD |
| Regurgitation area (RA cm^2^) | 2.6 | 2.1 | 2.0 | 2.4 | 3.2 | 1.8 | 2.8 | 2.3 | 2.4 | 1.9 | 2.6 | 2.5 | 2.1 | 2.4 | 0.4 |
| left atrial area (LA A , cm^2^) | 6.3 | 5.9 | 6.2 | 6.8 | 6.2 | 6.3 | 5.9 | 6.1 | 6.5 | 6.3 | 6.2 | 6.1 | 6.6 | 6.3 | 0.3 |
| RA/LAA | 0.4 | 0.4 | 0.3 | 0.4 | 0.5 | 0.3 | 0.5 | 0.4 | 0.4 | 0.3 | 0.4 | 0.4 | 0.3 | 0.4 | 0.1 |
| Regurgitation volume (RV ml) | 2.3 | 1.7 | 2.6 | 1.8 | 1.2 | 2.2 | 1.9 | 1.8 | 3.0 | 2.2 | 1.3 | 1.1 | 1.5 | 1.9 | 0.6 |
| Regurgitation fraction (RF %) | 38.7 | 39.0 | 40.0 | 36.2 | 35.7 | 43.1 | 38.7 | 39.3 | 42.2 | 36.7 | 35.8 | 38.2 | 39.1 | 38.7 | 2.3 |
| Regurgitation velocity (m/s) | 324.0 | 318.0 | 375.0 | 335.0 | 338.0 | 315.0 | 310.0 | 318.0 | 327.0 | 332.0 | 338.0 | 331.0 | 349.0 | 331.5 | 17.0 |
| LVEDV (ml) | 29.9 | 32.1 | 30.8 | 32.4 | 30.8 | 32.9 | 25.6 | 32.4 | 29.8 | 31.9 | 28.6 | 33.1 | 30.2 | 30.8 | 2.1 |
| LVESV (ml) | 8.8 | 9.2 | 7.0 | 6.9 | 7.9 | 9.1 | 6.9 | 8.2 | 7.7 | 7.5 | 6.8 | 7.4 | 7.2 | 7.7 | 0.8 |
| EF (%) | 76.7 | 73.8 | 62.4 | 76.8 | 79.6 | 74.2 | 76.6 | 77.6 | 68.9 | 77.6 | 71.5 | 80.1 | 62.3 | 73.7 | 5.9 |
| E/A | 1.3 | 1.7 | 1.8 | 2.5 | 1.2 | 1.9 | 2.1 | 1.2 | 2.3 | 2.8 | 1.7 | 2.2 | 0.7 | 1.8 | 0.6 |
| LAEDV (ml) | 21.5 | 27.7 | 22.3 | 26.3 | 23.5 | 25.6 | 23.6 | 21.3 | 21.6 | 24.8 | 27.3 | 26.3 | 23.5 | 24.3 | 2.2 |
| LAESV (ml) | 9.0 | 8.7 | 8.6 | 9.1 | 9.0 | 8.8 | 8.7 | 9.3 | 8.9 | 9.1 | 8.5 | 9.0 | 8.9 | 8.9 | 0.2 |
