# Supplementary material for: A Pig Model of Ischemic Mitral Regurgitation Induced by Mitral Chordae Tendinae Rupture and Implantation of an Ameroid Constrictor
Source: PLoS One. 2014 Dec 5;9(12):e111689. doi: 10.1371/journal.pone.0111689 (PMC4257529; doi:10.1371/journal.pone.0111689)
Supplement: Table S7 — Cardiac dimensions, function and regurgitation parameters two weeks after surgery in operated pig heart. (DOC) [file pone.0111689.s007.doc]

**Table S7 Cardiac dimensions, function and regurgitation parameters two weeks after surgery in operated pig heart**

|  | pig 1 | pig 2 | pig 3 | pig 4 | pig 5 | pig 6 | pig 7 | pig 8 | pig 9 | pig 10 | pig 11 | pig 12 | pig 13 | mean | SD |
| --- | --- | --- | --- | --- | --- | --- | --- | --- | --- | --- | --- | --- | --- | --- | --- |
|  |  |  |  |  |  |  |  |  |  |  |  |  |  |  |  |
| Regurgitation area (RA cm2) | 2.3 | 2.9 | 2.6 | 2.7 | 2.9 | 1.9 | 2.5 | 2.6 | 2.1 | 2.5 | 2.9 | 2.8 | 2.7 | 2.6 | 0.3 |
| left atrial area (LA A , cm2) | 8.2 | 9.2 | 7.8 | 8.5 | 8.5 | 8.3 | 8.6 | 8.4 | 7.9 | 8.5 | 8.3 | 8.2 | 7.9 | 8.3 | 0.4 |
| RA/LAA | 0.3 | 0.3 | 0.3 | 0.3 | 0.3 | 0.2 | 0.3 | 0.3 | 0.3 | 0.4 | 0.3 | 0.3 | 0.3 | 0.3 | 0.0 |
| Regurgitation volume (RV ml) | 3.7 | 2.8 | 8.5 | 5.2 | 8.3 | 7.5 | 5.7 | 4.5 | 3.8 | 9.7 | 4.8 | 3.5 | 1.2 | 5.3 | 2.5 |
| Regurgitation fraction (RF %) | 43.5 | 42.1 | 41.2 | 40.8 | 43.5 | 43.8 | 41.9 | 42.5 | 40.6 | 42.5 | 43.4 | 44.1 | 42.5 | 42.5 | 1.1 |
| Regurgitation velocity (m/s) | 275.0 | 335.0 | 331.0 | 295.0 | 365.0 | 348.0 | 297.0 | 346.0 | 357.0 | 296.0 | 315.0 | 351.0 | 315.0 | 325.1 | 28.3 |
| LVEDV (ml) | 32.4 | 38.0 | 39.5 | 38.5 | 37.8 | 38.5 | 42.0 | 37.3 | 38.5 | 37.9 | 30.0 | 43.2 | 38.4 | 37.8 | 3.4 |
| LVESV (ml) | 9.9 | 10.7 | 9.8 | 9.6 | 9.7 | 9.9 | 8.9 | 9.5 | 10.2 | 8.8 | 9.9 | 9.6 | 9.0 | 9.7 | 0.5 |
| EF (%) | 75.9 | 72.7 | 70.8 | 74.3 | 73.8 | 78.2 | 74.7 | 73.8 | 77.1 | 76.3 | 73.2 | 77.2 | 71.9 | 74.6 | 2.2 |
| E/A | 1.3 | 1.2 | 1.7 | 1.0 | 1.8 | 1.2 | 0.9 | 1.1 | 1.2 | 2.1 | 1.9 | 1.3 | 0.8 | 1.3 | 0.4 |
| LAEDV (ml) | 23.6 | 26.3 | 23.2 | 26.9 | 25.3 | 26.0 | 24.4 | 22.4 | 22.5 | 26.5 | 25.6 | 26.2 | 23.4 | 24.8 | 1.6 |
| LAESV (ml) | 9.8 | 8.9 | 9.3 | 10.1 | 9.9 | 9.7 | 9.3 | 9.7 | 9.3 | 8.9 | 8.8 | 9.6 | 9.9 | 9.5 | 0.4 |
