# Supplementary material for: A Pig Model of Ischemic Mitral Regurgitation Induced by Mitral Chordae Tendinae Rupture and Implantation of an Ameroid Constrictor
Source: PLoS One. 2014 Dec 5;9(12):e111689. doi: 10.1371/journal.pone.0111689 (PMC4257529; doi:10.1371/journal.pone.0111689)
Supplement: Table S8 — Cardiac dimensions, function and regurgitation parameters four weeks after surgery in operated pig heart. (DOC) [file pone.0111689.s008.doc]

**Table S8 Cardiac dimensions, function and regurgitation parameters four weeks after surgery in operated pig heart**

|  | pig 1 | pig 2 | pig 3 | pig 4 | pig 5 | pig 6 | pig 7 | pig 8 | pig 9 | pig 10 | pig 11 | pig 12 | pig 13 | mean | SD |
| --- | --- | --- | --- | --- | --- | --- | --- | --- | --- | --- | --- | --- | --- | --- | --- |
| Regurgitation area (RA cm2) | 2.6 | 2.4 | 2.7 | 1.9 | 3.6 | 1.7 | 2.9 | 2.8 | 2.4 | 2.1 | 2.8 | 3.2 | 1.4 | 2.5 | 0.6 |
| left atrial area (LA A , cm2) | 8.4 | 9.6 | 8.8 | 9.5 | 8.8 | 8.5 | 8.4 | 8.7 | 8.2 | 8.6 | 8.5 | 8.3 | 8.1 | 8.6 | 0.5 |
| RA/LAA | 0.3 | 0.3 | 0.3 | 0.3 | 0.3 | 0.2 | 0.3 | 0.3 | 0.3 | 0.4 | 0.3 | 0.2 | 0.3 | 0.3 | 0.0 |
| Regurgitation volume (RV ml) | 3.7 | 2.8 | 8.5 | 5.2 | 8.3 | 7.5 | 5.7 | 4.5 | 3.8 | 9.7 | 4.8 | 3.5 | 1.2 | 5.3 | 2.5 |
| Regurgitation fraction (RF %) | 48.8 | 45.2 | 49.3 | 49.8 | 43.7 | 46.6 | 43.9 | 41.5 | 45.6 | 44.5 | 46.6 | 48.2 | 49.3 | 46.4 | 2.6 |
| Regurgitation velocity (m/s) | 320.0 | 309.0 | 343.0 | 321.0 | 341.0 | 336.0 | 309.0 | 344.0 | 328.0 | 318.0 | 341.0 | 329.0 | 347.0 | 329.7 | 13.4 |
| LVEDV (ml) | 43.9 | 42.0 | 45.8 | 46.5 | 49.9 | 43.1 | 47.9 | 41.3 | 41.8 | 45.5 | 42.9 | 48.8 | 43.5 | 44.8 | 2.8 |
| LVESV (ml) | 14.2 | 16.1 | 15.5 | 16.3 | 17.6 | 14.1 | 14.2 | 15.5 | 16.3 | 15.2 | 16.2 | 12.6 | 14.1 | 15.2 | 1.3 |
| EF (%) | 64.6 | 57.3 | 62.8 | 58.8 | 65.6 | 78.0 | 68.7 | 66.3 | 79.7 | 68.2 | 55.2 | 75.5 | 62.3 | 66.4 | 7.7 |
| E/A | 1.5 | 2.3 | 1.1 | 0.9 | 1.2 | 2.5 | 1.2 | 2.5 | 1.1 | 2.4 | 1.1 | 1.5 | 2.6 | 1.7 | 0.7 |
| LAEDV (ml) | 33.2 | 32.3 | 30.8 | 29.4 | 35.2 | 32.0 | 29.6 | 32.1 | 29.5 | 33.3 | 35.1 | 35.1 | 33.4 | 32.4 | 2.1 |
| LAESV (ml) | 11.3 | 10.8 | 12.5 | 10.1 | 13.2 | 13.4 | 11.9 | 11.7 | 12.6 | 13.2 | 11.7 | 10.1 | 12.9 | 12.0 | 1.1 |
