# Supplementary material for: A Pig Model of Ischemic Mitral Regurgitation Induced by Mitral Chordae Tendinae Rupture and Implantation of an Ameroid Constrictor
Source: PLoS One. 2014 Dec 5;9(12):e111689. doi: 10.1371/journal.pone.0111689 (PMC4257529; doi:10.1371/journal.pone.0111689)
Supplement: Table S9 — Cardiac dimensions, function and regurgitation parameters eight weeks after surgery in operated pig heart. (DOC) [file pone.0111689.s009.doc]

**Table S9 Cardiac dimensions, function and regurgitation parameters eight weeks after surgery in operated pig heart**

|  | pig 1 | pig 2 | pig 3 | pig 4 | pig 5 | pig 6 | pig 7 | pig 8 | pig 9 | pig 10 | pig 11 | pig 12 | pig 13 | mean | SD |
| --- | --- | --- | --- | --- | --- | --- | --- | --- | --- | --- | --- | --- | --- | --- | --- |
| Regurgitation area (RA cm2) | 2.8 | 2.7 | 2.6 | 3.8 | 2.6 | 1.6 | 3.7 | 3.9 | 1.5 | 2.7 | 2.9 | 3.6 | 1.5 | 2.8 | 0.8 |
| left atrial area (LA A , cm2) | 8.5 | 9.2 | 9.6 | 9.4 | 9.5 | 7.3 | 11.4 | 12.7 | 7.4 | 13.1 | 6.5 | 9.3 | 6.1 | 9.2 | 2.2 |
| RA/LAA | 0.3 | 0.3 | 0.3 | 0.4 | 0.3 | 0.2 | 0.3 | 0.3 | 0.2 | 0.2 | 0.4 | 0.4 | 0.2 | 0.3 | 0.1 |
| Regurgitation volume (RV ml) | 4.7 | 3.6 | 7.5 | 5.8 | 8.2 | 8.3 | 6.7 | 3.8 | 4.2 | 9.2 | 4.7 | 6.6 | 7.4 | 6.2 | 1.9 |
| Regurgitation fraction (RF %) | 49.7 | 46.2 | 47.4 | 50.6 | 48.2 | 45.9 | 53.2 | 52.3 | 53.2 | 42.2 | 54.2 | 49.2 | 48.2 | 49.3 | 3.5 |
| Regurgitation velocity (m/s) | 428.0 | 209.0 | 555.0 | 536.0 | 379.0 | 286.0 | 559.0 | 487.0 | 217.0 | 498.0 | 216.0 | 489.0 | 527.0 | 414.3 | 136.8 |
| LVEDV (ml) | 53.7 | 44.0 | 46.5 | 52.5 | 48.6 | 53.2 | 46.3 | 45.2 | 43.8 | 43.2 | 48.6 | 54.2 | 55.8 | 48.9 | 4.5 |
| LVESV (ml) | 16.2 | 17.6 | 16.1 | 17.1 | 17.6 | 15.2 | 15.6 | 16.2 | 17.9 | 17.2 | 18.3 | 13.3 | 14.1 | 16.3 | 1.5 |
| EF (%) | 63.1 | 58.1 | 60.9 | 54.6 | 61.8 | 68.0 | 66.3 | 64.1 | 77.8 | 67.8 | 60.7 | 75.3 | 66.8 | 65.0 | 6.4 |
| E/A | 2.4 | 1.9 | 1.2 | 1.0 | 0.8 | 2.6 | 1.2 | 2.5 | 1.3 | 2.6 | 1.1 | 1.2 | 1.4 | 1.6 | 0.7 |
| LAEDV (ml) | 35.6 | 32.4 | 29.7 | 37.2 | 39.3 | 35.0 | 31.2 | 34.2 | 31.6 | 29.2 | 38.9 | 40.4 | 38.5 | 34.9 | 3.8 |
| LAESV (ml) | 13.3 | 12.1 | 14.2 | 10.9 | 15.2 | 14.5 | 12.3 | 12.2 | 13.5 | 14.3 | 13.2 | 12.1 | 13.2 | 13.2 | 1.2 |
